# Head-to-head comparison of subcutaneous abatacept versus adalimumab for rheumatoid arthritis: Findings of a phase IIIb, multinational, prospective, randomized study

**DOI:** 10.1002/art.37711

**Published:** 2013-01

**Authors:** Michael E Weinblatt, Michael Schiff, Robert Valente, Désirée van der Heijde, Gustavo Citera, Cathy Zhao, Michael Maldonado, Roy Fleischmann

**Affiliations:** 1 Brigham and Women's HospitalBoston, Massachusetts; 2 University of ColoradoDenver; 3 Arthritis Center of NebraskaLincoln; 4 Leiden University Medical CenterLeiden, The Netherlands; 5 Instituto de Rehabilitación Psicofísica de Buenos AiresBuenos Aires, Argentina; 6 Bristol-Myers SquibbPrinceton, New Jersey; 7 University of Texas Southwestern Medical CenterDallas

## Abstract

**Objective:**

There is a need for comparative studies to provide evidence-based treatment guidance for biologic agents in rheumatoid arthritis (RA). Therefore, this study was undertaken as the first head-to-head comparison of subcutaneous (SC) abatacept and SC adalimumab, both administered along with background methotrexate (MTX), for the treatment of RA.

**Methods:**

Patients with active RA who were naive to treatment with biologic agents and had an inadequate response to MTX were randomly assigned to receive 125 mg SC abatacept weekly or 40 mg SC adalimumab biweekly, both given in combination with MTX, in a 2-year study. The primary end point was treatment noninferiority, assessed according to the American College of Rheumatology 20% improvement response (ACR20) at 1 year.

**Results:**

Of the 646 patients who were randomized and treated, 86.2% receiving SC abatacept and 82% receiving SC adalimumab completed 12 months of treatment. At 1 year, 64.8% of patients in the SC abatacept group and 63.4% in the SC adalimumab group demonstrated an ACR20 response; the estimated difference between groups was 1.8% (95% confidence interval −5.6%, 9.2%), thus demonstrating the noninferiority of abatacept compared to adalimumab. All efficacy measures showed similar results and kinetics of response between treatments. The rate of radiographic nonprogression (defined as a total modified Sharp/van der Heijde score [SHS] less than or equal to the smallest detectable change) was 84.8% for SC abatacept–treated patients and 88.6% for SC adalimumab–treated patients, while the mean change from baseline in the total SHS was 0.58 and 0.38, respectively. In the SC abatacept and SC adalimumab groups, the incidence of serious adverse events (SAEs) was 10.1% and 9.1%, respectively, and the rate of serious infections was 2.2% and 2.7%, respectively. In patients treated with SC abatacept, the frequency of discontinuations due to AEs was 3.5% and discontinuations due to SAEs was 1.3%, while in patients treated with SC adalimumab, the frequencies were 6.1% and 3%, respectively. Injection site reactions occurred in 3.8% of patients receiving SC abatacept compared to 9.1% of patients receiving SC adalimumab (*P* = 0.006).

**Conclusion:**

The results demonstrate that SC abatacept and SC adalimumab have comparable efficacy in patients with RA, as shown by similar kinetics of response and comparable inhibition of radiographic progression over 1 year of treatment. The safety was generally similar, other than the occurrence of significantly more local injection site reactions in patients treated with SC adalimumab.

The treatment of rheumatoid arthritis (RA) has evolved significantly over the last decade, with the early use of methotrexate (MTX) and the addition of targeted biologic disease-modifying antirheumatic drugs (bDMARDs) in patients with an incomplete response to MTX (1). The combination of bDMARDs and MTX as the anchor drug has demonstrated the best clinical outcomes in trials and routine practice, and has emerged as the de facto standard of care for patients with moderate-to-severe disease (1, 2).

Tumor necrosis factor inhibitors (TNFi) were the first approved bDMARDs for RA, followed by 4 additional bDMARD therapies with different mechanisms of action (1, 3). TNFi have become the most widely used initial bDMARD. Adalimumab, a TNFi, and abatacept, a T cell costimulation modulator, have been extensively studied in RA, and treatment of RA patients with these agents, in combination with MTX, in randomized trials has produced similar clinical results (4–11). With multiple therapeutic options available for the treatment of RA (12), a critical question is whether bDMARDs with different mechanisms of action have comparable clinical efficacy, comparable safety, and similar effects on inhibition of radiographic progression. To date, there has been no head-to-head comparison of bDMARDs in an RA clinical trial (13). These trials are essential for evidence-based treatment decisions (1, 14).

AMPLE (Abatacept versus Adalimumab Comparison in Biologic-Naive RA Subjects with Background Methotrexate) is a 2-year phase IIIB, multinational, prospective, randomized study. Since comparable responses to treatment have been demonstrated with both abatacept and adalimumab in separate clinical studies, a noninferiority design was utilized in this head-to-head study to determine the comparative effects of these agents on clinical responses, radiographic activity, and overall safety (15). In this report, we present the first-year results of this study.

## PATIENTS AND METHODS

### Patients

Eligible patients met the American College of Rheumatology (ACR) 1987 classification criteria for RA (16), were at least 18 years of age, had a confirmed diagnosis of RA for ≤5 years, had an inadequate response to MTX, and had not received previous bDMARD therapy. At randomization, patients were required to have active disease, defined as a score of ≥3.2 on the Disease Activity Score in 28 joints using the C-reactive protein level (DAS28-CRP) (17), as well as a history of one or both of the following features: 1) seropositivity for anti–cyclic citrullinated peptide antibodies or rheumatoid factor, and/or 2) an elevated erythrocyte sedimentation rate (ESR) or CRP level.

### Study design and treatment administration

Patients were randomly assigned in a 1:1 ratio to receive 125 mg abatacept (Orencia; Bristol-Myers Squibb), administered SC once per week (without an intravenous loading dose), or 40 mg adalimumab (Humira; Abbott Laboratories), administered SC every other week, both given in combination with MTX. Patients were stratified by disease activity, according to those with high disease activity (defined as a DAS28-CRP score >5.1) and those with moderate disease activity (defined as a DAS28-CRP score ≥3.2 and ≤5.1).

A total of 120 sites from North and South America participated in this study, and ∼70% of the patients were from sites in the US. The first year of this study was completed in October 2011.

Patients were concomitantly treated with a stable dosage of MTX (between ≥15 mg/week and ≤25 mg/week, or at least ≥7.5 mg/week in patients with documented intolerance to higher doses). In addition, patients were allowed to receive either hydroxychloroquine or sulfasalazine; other DMARDs were not allowed during the study. Stable, low-dose oral corticosteroids (≤10 mg/day prednisone equivalent) were permitted. Up to 2 courses of high-dose corticosteroids (such as a short [defined as a maximum of 2 weeks] oral course of high-dose corticosteroids, a single intramuscular dose of corticosteroid, or a single intraarticular injection of corticosteroid) were permitted, except within 42 days of day 365. Use of nonsteroidal antiinflammatory drugs (NSAIDs), including aspirin, was permitted, provided that the dosage was stable; additional NSAIDs were not allowed within 12 hours before a clinical assessment.

Double-blinding for the study drugs was not feasible, due to logistic barriers that did not permit masking of the adalimumab syringes. Patients were not blinded with regard to their study drug. Clinical assessors were blinded with regard to each patient's treatment. These blinded assessors evaluated the patients' joints, assessed disease activity, and defined adverse event (AE) causality. In addition, different physicians reviewed and approved all of the data entry forms but did not contribute to the data collection. Radiographs were scored by independent readers who were blinded with regard to the treatment regimen.

The protocol was approved by the institutional review boards and independent ethics committees at the participating sites, and the study was conducted in accordance with the Declaration of Helsinki and was consistent with the International Conference on Harmonisation and Good Clinical Practice. All patients provided written informed consent before randomization. The protocol was jointly developed by the academic authors and the study sponsor.

### Efficacy assessments

The primary outcome measure for determining the noninferiority of SC abatacept compared to SC adalimumab was the proportion of patients in each group achieving a 20% response on the ACR improvement criteria (ACR20) at 1 year (18). Efficacy assessments were performed on days 1, 15, and 29, and every 4 weeks thereafter. An ACR20 response indicates a decrease of at least 20% in the number of both tender and swollen joints (68 joints assessed for tenderness, and 66 joints assessed for swelling) as well as at least a 20% improvement in at least 3 of the following features: the patient's global assessment of disease activity (on a 100-mm visual analog scale [VAS]), the patient's assessment of pain (on a 100-mm VAS), the patient's assessment of physical function on the Health Assessment Questionnaire Disability Index (HAQ DI) (19), the physician's global assessment of disease activity (on a 100-mm VAS), and the CRP level.

Secondary end points included a 50% and 70% level of improvement according to the ACR response criteria (ACR50 and ACR70), changes in the DAS28-CRP score, and achievement of clinical remission (defined as a DAS28-CRP score of <2.6), low disease activity (defined as a DAS28-CRP score of ≤3.2), and improvement in physical function (defined as a change from baseline in the HAQ DI of ≥0.3 units) (20, 21). In addition, scores on the Routine Assessment of Patient Index Data 3 (RAPID-3) (on a scale of 0–10) were calculated (22). Patient's assessment of the severity of fatigue over the past week was determined using a 100-mm VAS (23).

Plain radiographs of the hands and feet were obtained from all patients on day 1 (baseline) and day 365 (1 year), and scored for radiographic damage using the modified Sharp/ van der Heijde scoring system (SHS) (24). Radiographs were centrally evaluated by readers who were blinded with regard to the drug treatment and the sequence of films. Rates of radiographic nonprogression were determined using the smallest detectable change (SDC), an estimate of the measurement error of simultaneously read films (25), in which a total SHS score less than or equal to the SDC was used as the cutoff for defining radiographic nonprogression from baseline to 1 year. Interreader and intrareader variability was also assessed by intraclass correlations. Mean changes from baseline in the total SHS score, erosion score, and joint space narrowing score were calculated at the end of year 1. In addition, cumulative probability plots were used to assess the distribution of scores from individual patients.

### Safety and autoantibody assessments

Safety events were classified using the Medical Dictionary for Regulatory Activities (MedDRA) (version 14.1). Both the severity of AEs and their relationship to the study treatment were noted by 2 investigators (RV and GC). Local injection site reactions and autoimmune events were prespecified based on a list of MedDRA Preferred Terms.

Blood samples for measurement of autoantibodies (antinuclear antibodies [ANAs] and anti–double-stranded DNA [anti-dsDNA] autoantibodies) were collected at baseline and 1 year. Samples were first tested for ANAs by indirect immunofluorescence assay using a HEp-2 cell line substrate. Samples positive for ANAs were then further tested for anti-dsDNA by indirect immunofluorescence assay using a *Crithidia luciliae* substrate.

### Statistical analysis

All efficacy and safety analyses were performed using the intent-to-treat (ITT) population, which included all patients who were randomized and received at least one dose of study drug. A per-protocol population, which included the subset of patients from the ITT population who had no relevant protocol deviations, was used to confirm the primary efficacy end point. All patients who prematurely discontinued the study after receiving the study drug, regardless of the reason, were considered nonresponders at all scheduled visits subsequent to the point of discontinuation.

Based on assumption of a 2.5% one-sided level of significance with 93% power to detect a difference between groups and a 12% noninferiority margin, a sample size of 648 patients equally randomized to the 2 treatment groups was needed to test the hypothesis of treatment noninferiority. The 12% noninferiority margin preserves at least 50% of the treatment effect, according to results from previous studies (5, 8, 9). This allows for a maximum difference of −4.7% (95% confidence interval [95% CI] −12%, 2.6%) in the ACR20 response between SC abatacept and SC adalimumab, a difference that was considered to be clinically meaningful. A 5% protocol-deviation rate would maintain at least 90% power to test the primary end point with the per-protocol population.

The proportion of patients with local injection site reactions from baseline to 1 year was a key secondary end point. Statistical testing for this secondary end point was prespecified if the primary end point (noninferiority according to the ACR20 improvement response) was met.

Baseline demographic and clinical characteristics were analyzed descriptively for all patients. For the response rates determined in efficacy analyses, the estimate of the proportion of responders (with 95% CIs) within each treatment group, as well as the 95% CIs for the difference in response rates between treatment groups, are presented. For assessments of change from baseline, analyses of covariance were used, with treatment assignment serving as the main factor and baseline values and disease activity group as covariates. Point estimates and 95% CIs are provided for the difference in adjusted mean change from baseline between the 2 treatment groups. For the mean change in DAS28-CRP scores, missing values were imputed using a last observation carried forward analysis (excluding patients for whom only baseline observations were available). Radiographs were obtained at baseline and at 1 year. For patients who were unwilling or unable to return after 1 year, radiographs were obtained at the time of discontinuation. In these patients, the 1-year data were imputed using linear extrapolation of the scores for radiographs obtained at the time of discontinuation. Data on safety parameters in each treatment group are expressed as frequencies.

## RESULTS

### Disposition of patients

**Figure 1:**
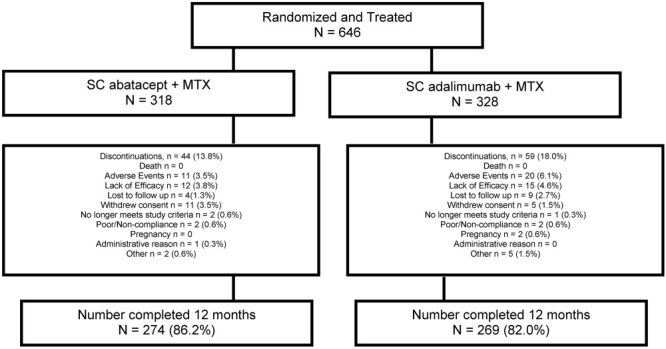
Disposition of rheumatoid arthritis patients in the intent-to-treat population randomized to receive either subcutaneous (SC) abatacept or SC adalimumab, both given in combination with methotrexate (MTX), over 1 year.

In total, 646 patients were randomized and treated (318 in the SC abatacept group and 328 in the SC adalimumab group) (Figure 1). At 1 year, 86.2% (274 of 318) of the SC abatacept–treated patients and 82% (269 of 328) of the SC adalimumab–treated patients completed the study. The main reasons for discontinuation were lack of efficacy (3.8% of SC abatacept–treated patients versus 4.6% of SC adalimumab–treated patients) and AEs (3.5% of SC abatacept–treated patients versus 6.1% of SC adalimumab–treated patients).

### Baseline demographic and clinical characteristics

The demographic and clinical characteristics of the patients at baseline were balanced across the SC abatacept and SC adalimumab groups and were typical for RA studies (Table 1). The mean age of the patients in each group was ∼51 years, and the mean disease duration was 1.9 years in the SC abatacept group and 1.7 years in the SC adalimumab group. The mean ± SD DAS28-CRP score at baseline was 5.5 ± 1.1 in both groups, with an equal proportion of patients with moderate disease activity and patients with high disease activity in each group.

**Table 1:** Baseline demographic and clinical characteristics of the patients in the intent-to-treat population*

	SC abatacept + MTX (n = 318)	SC adalimumab + MTX (n = 328)
Age, years	51.4 ± 12.6	51.0 ± 12.8
Weight, kg	80.8 ± 20.3	80.1 ± 20.7
Female, %	81.4	82.3
White, %	80.8	78.0
Duration of disease, years	1.9 ± 1.4	1.7 ± 1.4
Geographic region, no. (%)		
North America	230 (72.3)	235 (71.6)
South America	88 (27.7)	93 (28.4)
No. of tender joints†	25.4 ± 15.3	26.3 ± 15.8
No. of swollen joints†	15.8 ± 9.8	15.9 ± 10.0
HAQ DI score‡	1.5 ± 0.7	1.5 ± 0.7
Pain score§	63.1 ± 22.3	65.5 ± 21.8
Global assessment of disease activity§		
Patient's	61.1 ± 22.1	61.5 ± 22.5
Physician's	58.8 ± 18.6	58.8 ± 18.9
CRP, mg/dl	1.6 ± 2.1	1.5 ± 2.8
DAS28-CRP score¶	5.5 ± 1.1	5.5 ± 1.1
Positive for RF, no. (%)#	240 (75.5)	254 (77.4)
MTX dosage, mg/week	17.5 ± 6.35	17.3 ± 6.16
Concomitant medication		
Corticosteroids, %	50.9	50.3
Prednisone dose, mg/day	6.6 ± 2.59	6.4 ± 2.67
Sulfasalazine, %	3.1	3.4
Hydroxychloroquine, %	13.2	10.7
Radiographic findings		
Total SHS (scale 0–448)	24.8 ± 37.1	24.2 ± 32.9
Estimated annual SHS	25.1 ± 64.9	22.4 ± 41.7

* Except where indicated otherwise, values are the mean ± SD. SC = subcutaneous; MTX = methotrexate; RF = rheumatoid factor; SHS = modified Sharp/van der Heijde score (of radiographic damage).

† A total of 68 joints were assessed for tenderness and 66 were assessed for swelling.

‡ The degree of disability was assessed with the Health Assessment Questionnaire Disability Index (HAQ DI), in which scores range from 0 to 3, with higher scores indicating greater disability.

§ A 100-mm visual analog scale was used, in which higher values indicate more severe pain or abnormalities.

¶ Arthritis disease activity was assessed with the Disease Activity Score in 28 joints using the C-reactive protein level (DAS28-CRP), in which scores range from 0 to 9.31, with higher scores indicating more severe disease activity.

# Information was collected from medical records at the time of screening (not based on testing at screening).

### Concomitant medications

The proportions of patients taking concomitant medications at baseline are shown in Table 1. The mean ± SD dosage of MTX was 17.5 ± 6.35 mg/week in the SC abatacept group and 17.3 ± 6.16 mg/week in the SC adalimumab group; 86.8% of the SC abatacept–treated patients and 82% of the SC adalimumab–treated patients were reported to have been taking MTX for at least 3 months. The mean dose of oral prednisone was 6.6 mg/day in the SC abatacept group and 6.4 mg/day in the SC adalimumab group. A small proportion of patients treated with either SC abatacept or SC adalimumab were taking allowable DMARDs at the time of enrollment (3.1% versus 3.4% taking sulfasalazine, and 13.2% versus 10.7% taking hydroxychloroquine). The proportions of patients receiving concomitant corticosteroids over 1 year were 62.6% (SC abatacept) and 61.0% (SC adalimumab). More than 1 course of high-dose corticosteroids was received by 2.8% of the SC abatacept–treated patients and 1.2% of the SC adalimumab–treated patients.

### Clinical efficacy

The proportion of patients achieving an ACR20 response at 1 year was 64.8% (95% CI 59.5%, 70.0%) in the SC abatacept group and 63.4% (95% CI 58.2%, 68.6%) in the SC adalimumab group; the estimate of the difference in ACR20 response rates between groups was 1.8% (95% CI −5.6%, 9.2%), thus demonstrating the noninferiority of SC abatacept compared to SC adalimumab. The estimate of difference in the ACR20 response rates between groups in the per-protocol population was 1.1% (95% CI −6.5%, 8.7%), which confirms the noninferiority of SC abatacept compared to SC adalimumab.

**Figure 2:**
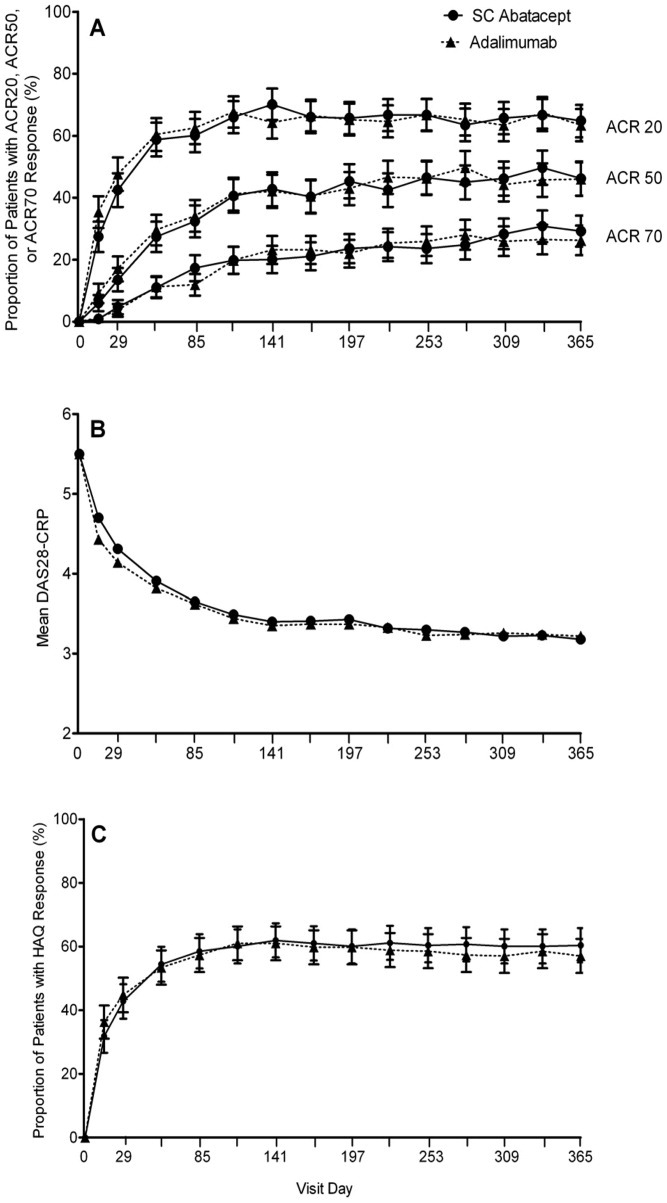
Proportions of rheumatoid arthritis patients meeting efficacy criteria in the subcutaneous (SC) abatacept or SC adalimumab treatment groups over 1 year. A, Rates of American College of Rheumatology 20%, 50%, and 70% improvement responses (ACR20, ACR50, and ACR70) over 1 year are shown for each treatment group (intent-to-treat population; n = 318 SC abatacept, n = 328 SC adalimumab). Bars show the mean and 95% confidence interval. B, Disease activity was measured over 1 year in each treatment group with the Disease Activity Score in 28 joints using the C-reactive protein level (DAS28-CRP). Mean values are shown. C, Physical function was assessed over 1 year in each treatment group using patients' responses on the Health Assessment Questionnaire Disability Index (HAQ DI) (improvement defined as an increase of ≥0.3 units from baseline). Bars show the mean and 95% confidence interval.

The ACR20, ACR50, and ACR70 response rates over 1 year are shown in Figure 2A. The kinetics of response were similar between the 2 groups. On day 29, 42.5% (95% CI 37.0%, 47.9%) of patients in the SC abatacept group and 47.6% (95% CI 42.2%, 53.0%) of patients in the SC adalimumab group had achieved an ACR20 response, and these response rates remained comparable between the 2 groups for the duration of the study (Figure 2A). At 1 year, 46.2% (95% CI 40.7%, 51.7%) and 46% (95% CI 40.6%, 51.4%) of SC abatacept– and SC adalimumab–treated patients, respectively, had achieved an ACR50 response, and 29.2% (95% CI 24.2%, 34.2%) and 26.2% (95% CI 21.5%, 31.0%), respectively, had achieved an ACR70 response.

The mean DAS28-CRP scores over time are shown in Figure 2B. At 1 year, the adjusted mean improvement from baseline in the DAS28-CRP score was −2.30 ± 0.08 (mean ± SEM) in the SC abatacept group and −2.27 ± 0.08 in the SC adalimumab group. Similar improvements in the level of disease activity were observed between the 2 treatment groups at 1 year, with 59.3% (95% CI 53.5%, 65.1%) of SC abatacept–treated patients and 61.4% (95% CI 55.6%, 67.3%) of SC adalimumab–treated patients exhibiting low disease activity (defined as a DAS28-CRP score of ≤3.2).

When remission of RA from baseline to year 1 was assessed using different measures (the DAS28-CRP, the Clinical Disease Activity Index [CDAI] [26], and the Simplified Disease Activity Index [SDAI] [27]), the proportions of patients achieving disease remission were similar in the SC abatacept and SC adalimumab treatment groups (with the DAS28-CRP, 43.3% [95% CI 37.4%, 49.1%] versus 41.9% [95% CI 36.0%, 47.9%]; with the CDAI, 23.5% [95% CI 18.5%, 28.5%] versus 24.0% [95% CI 18.8%, 29.1%]; and with the SDAI, 23.3% [95% CI 18.3%, 28.3%] versus 24.8% [95% CI 19.6%, 30.0%]). In a post hoc analysis, the remission rate based on the ACR/European League Against Rheumatism (EULAR) proposed Boolean-based definition of remission (28) was determined at 1 year in all patients without missing data. The rate of remission according to the ACR/EULAR Boolean-based definition (based on swollen/tender joint counts from a total of 66/68 joints assessed) was 13.5% (95% CI 9.4%, 17.5%) in the SC abatacept treatment group (n = 37) and 15.7% (95% CI 11.4%, 20.1%) in the SC adalimumab treatment group (n = 42).

Improvements in the HAQ DI score were comparable between treatment groups. On day 29, 42.8% (95% CI 37.3%, 48.2%) of patients in the SC abatacept group and 44.8% (95% CI 39.4%, 50.2%) of patients in the SC adalimumab group were HAQ DI responders, which, by the end of year 1, had increased to 60.4% (95% CI 55.0%, 65.8%) and 57.0% (95% CI 51.7%, 62.4%), respectively (Figure 2C) (estimate of difference between groups 3.4% [95% CI −4.5%, 11.3%]). The adjusted mean change in the HAQ DI score from baseline to 1 year was −0.60 ± 0.04 (mean ± SEM) in the SC abatacept group and −0.59 ± 0.03 in the SC adalimumab group.

**Figure 3:**
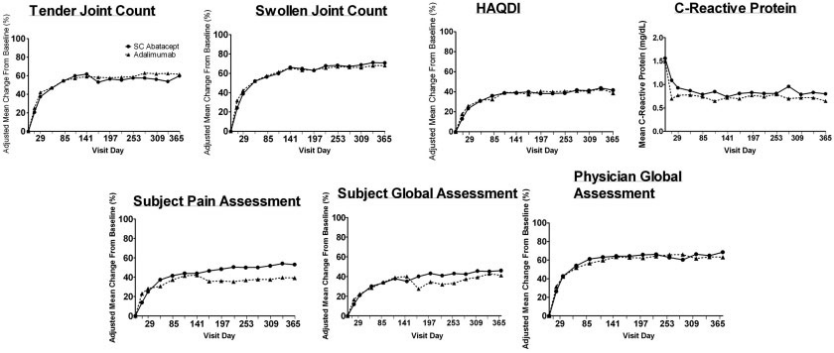
The 7 components of the American College of Rheumatology core set of outcome measures were assessed in patients treated with subcutaneous (SC) abatacept or SC adalimumab over 1 year. Results are the adjusted mean change from baseline to 1 year. HAQ DI = Health Assessment Questionnaire Disability Index

Similar changes over time were seen in components of the ACR core set of outcome measures (29) in each group, in both physician- and patient-reported outcomes. Figure 3 shows the changes in the 7 components from baseline to 1 year in both treatment groups. During 1 year of treatment, while similar trends were observed between the treatment groups for some components (tender and swollen joint counts, HAQ DI scores, and physician's global assessment of disease activity), different trends were seen for a few of the other components (patient's assessment of pain, patient's global assessment of disease activity, and CRP level).

At 1 year, the adjusted improvements (mean ± SEM) in each ACR component in patients treated with SC abatacept compared to patients treated with SC adalimumab were as follows: for improvement in tender joint counts, 59.8 ± 4.0% versus 61.4 ± 4.0% (adjusted mean difference −1.6% [95% CI −12.4%, 9.1%]); for improvement in swollen joint counts, 70.9 ± 2.6% versus 68.2 ± 2.6% (adjusted mean difference 2.6% [95% CI −4.3%, 9.6%]); for improvement in the HAQ DI score, 41.7 ± 3.2% versus 38.7 ± 3.2% (adjusted mean difference 3.0% [95% CI −5.6%, 11.7%]); for improvement in physician's global assessment of disease activity, 68.5 ± 4.3% versus 63.0 ± 4.3% (adjusted mean difference −5.5% [95% CI −6.0%, 17.0%]); for improvement in patient's assessment of pain, 53 ± 6.1% versus 39.2 ± 6.0% (adjusted mean difference 13.8% [95% CI −2.5%, 30.1%]); and for improvement in patient's global assessment of disease activity, 46.1 ± 3.5% versus 41.2 ± 3.4% (adjusted mean difference 4.9% [95% CI −4.4%, 14.1%]). CRP levels (mean ± SD) were reduced from baseline to 1 year in both groups, to 0.80 ± 1.13 mg/dl in the SC abatacept group and 0.65 ± 1.21 mg/dl in the SC adalimumab group.

At the end of year 1, both treatment groups showed a similar improvement in RAPID-3 scores. The mean change from baseline in the RAPID-3 scores was −2.87 (95% CI −3.10, −2.63) in the SC abatacept group and −2.74 (95% CI −2.98, −2.51) in the SC adalimumab group, with an estimate of difference between groups of −0.12 (95% CI −0.44, 0.20). Furthermore, fatigue scores improved in both groups (mean change −23.2 in SC abatacept–treated patients versus −21.4 in SC adalimumab–treated patients; adjusted mean difference between groups −1.8 [95% CI −5.8, 2.2]).

### Radiographic nonprogression

**Figure 4:**
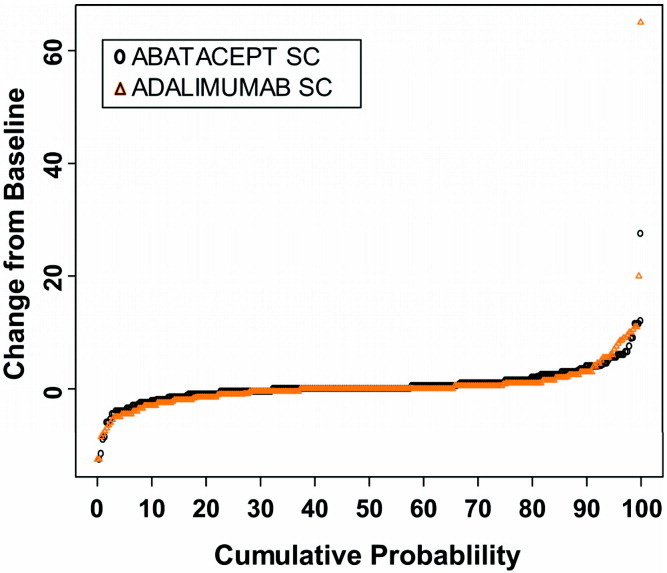
Radiographic outcomes in patients treated with subcutaneous (SC) abatacept or SC adalimumab over 1 year. The cumulative probability plot shows the distribution of change in total modified Sharp/van der Heijde scores of radiographic damage from baseline to 1 year by treatment group.

Paired radiographic images obtained at baseline and 1 year were available for 91.1% of patients in the SC abatacept group and 88.1% of patients in the SC adalimumab group. The cumulative probability plot with the distribution of change in the total SHS scores from baseline to 1 year showed that inhibition of radiographic damage was similar in both treatment groups (Figure 4). At 1 year, changes from baseline in radiographic disease features in each treatment group (mean ± SD) were as follows: for change in total SHS score, 0.58 ± 3.22 in SC abatacept–treated patients versus 0.38 ± 5 in SC adalimumab–treated patients; for change in erosion score, 0.29 ± 1.84 in SC abatacept–treated patients versus −0.01 ± 2.83 in SC adalimumab–treated patients; and for change in joint space narrowing score, 0.28 ± 1.92 in SC abatacept–treated patients versus 0.39 ± 2.50 in SC adalimumab–treated patients. The rate of radiographic nonprogression (defined as change from baseline in the total SHS score less than or equal to the SDC, at a cutoff level of 2.8) was observed to be 84.8% in the SC abatacept group and 88.6% in the SC adalimumab group (estimated difference between groups 4.1% [95% CI −1.5%, 9.6%]).

### Safety

Most safety outcomes in this study were comparable between the treatment groups. The rate of AEs and the rate of serious AEs (SAEs) were balanced between the groups (rate of SAEs, 10.1% of patients in the SC abatacept group versus 9.1% of patients in the SC adalimumab group) (Table 2). Rates of discontinuations due to AEs and discontinuations due to SAEs were low in both groups; the discontinuation rate in the SC adalimumab group was, however, almost twice the rate in the SC abatacept group (discontinuations due to AEs, 6.1% versus 3.5%; discontinuations due to SAEs, 3% versus 1.3%). One death due to sudden cardiac arrest occurred in a 66-year-old patient with a history of hypertension in the SC abatacept group; this event was considered by the investigator to be unrelated to the study treatment.

**Table 2:** Safety summary*

Event	SC abatacept + MTX (n = 318)	SC adalimumab + MTX (n = 328)
Deaths	1 (0.3)	0
SAEs	32 (10.1)	30 (9.1)
Related SAEs	8 (2.5)	11 (3.4)
Discontinued due to SAEs	4 (1.3)	10 (3.0)
Serious infections and infestations	7 (2.2)	9 (2.7)
Pneumonia	3 (0.9)	2 (0.6)
Urinary tract infection	2 (0.6)	0
Gastroenteritis	1 (0.3)	0
Helicobacter gastritis	1 (0.3)	0
Arthritis, bacterial	0	3 (0.9)
Chest wall abscess	0	1 (0.3)
Diverticulitis	0	1 (0.3)
Meningitis	0	1 (0.3)
Staphlyococcal bursitis	0	1 (0.3)
AEs	280 (88.1)	283 (86.3)
Related AEs	111 (34.9)	131 (39.9)
Discontinued due to AEs	11 (3.5)	20 (6.1)
Malignancies	5 (1.6)	4 (1.2)
Squamous cell carcinoma, skin	2 (0.6)	0
Diffuse large B cell lymphoma	1 (0.3)	0
Prostate cancer	1 (0.3)	0
Squamous cell carcinoma, lung	1 (0.3)	0
Basal cell carcinoma	0	2 (0.6)
Small cell lung cancer	0	1 (0.3)
Transitional cell carcinoma	0	1 (0.3)
Autoimmune events	10 (3.1)	4 (1.2)
Psoriasis	3 (0.9)	1 (0.3)
Erythema nodosum	1 (0.3)	1 (0.3)
Leukocytoclastic vasculitis	1 (0.3)	0
Raynaud's phenomenon	2 (0.6)	1 (0.3)
Cutaneous lymphocytic vasculitis	1 (0.3)	0
Episcleritis	1 (0.3)	0
Sjögren's syndrome	1 (0.3)	0
Anti-dsDNA seropositivity	0	1 (0.3)
Local injection site reactions†	12 (3.8)	30 (9.1)
Hematoma	5 (1.6)	3 (0.9)
Pruritis	1 (0.3)	7 (2.1)
Erythema	2 (0.6)	14 (4.3)
Pain	0	8 (2.4)
Reaction	3 (0.9)	4 (1.2)

* Values are the number (%) of patients with events. SC = subcutaneous; MTX = methotrexate; SAEs = serious adverse events; anti-dsDNA = anti–double-stranded DNA.

† The 5 most common local injection site reactions are reported.

### Infections

Overall, 63.2% of the SC abatacept–treated patients and 61.3% of the SC adalimumab–treated patients had an infection during the treatment period, with nasopharyngitis and upper respiratory tract infections being the most frequently reported in each treatment group. Serious infections occurred in 7 patients in the SC abatacept group (2.2%) and 9 patients in the SC adalimumab group (2.7%) (Table 2). While none of the 7 serious infections observed in patients in the SC abatacept group (3 cases of pneumonia, 2 cases of urinary tract infection, 1 case of gastroenteritis, and 1 case of helicobacter gastritis) led to treatment discontinuation, 5 of the 9 serious infections in the SC adalimumab group (2 cases of pneumonia, 3 cases of bacterial arthritis, and 1 case each of chest wall abscess, diverticulitis, meningitis, and staphylococcal bursitis) led to treatment discontinuation. Two opportunistic infections, 1 in each treatment group, occurred during the first year of treatment; both were cases of mucocutaneous oral candidiasis that were recorded as AEs, and neither of these patients discontinued treatment. No cases of tuberculosis were observed. Herpes zoster infections were reported in both groups (in 4 SC abatacept–treated patients [1.2%] versus 3 SC adalimumab–treated patients [0.9%]).

### Malignancies

Malignancies occurred in 5 patients (1.6%) in the SC abatacept group (2 with squamous cell carcinoma of the skin, 1 with diffuse large B cell lymphoma, 1 with squamous cell carcinoma of the lung, and 1 with prostate cancer) and in 4 patients (1.2%) in the SC adalimumab group (2 with basal cell carcinoma, 1 with small cell lung cancer, and 1 with transitional cell carcinoma) (Table 2).

### Autoimmune events

Autoimmune events were reported in 10 patients (3.1%) in the SC abatacept group and 4 patients (1.2%) in the SC adalimumab group (Table 2). None of these events were considered serious, and only 2 led to discontinuation, 1 in each treatment group (1 case of psoriasis in the SC abatacept group and 1 case of anti-dsDNA seroconversion in the SC adalimumab group).

Fewer patients in the SC abatacept group than in the SC adalimumab group underwent autoantibody seroconversion from baseline to 1 year. Seropositivity for ANAs was observed in 12 SC abatacept–treated patients (5.2% [95% CI 2.4%, 8.1%]) compared to 28 SC adalimumab–treated patients (13.3% [95% CI 8.7%, 17.9%]), while anti-dsDNA antibodies were detected in 1 SC abatacept–treated patient (0.3% [95% CI −0.3%, 1.0%]) compared to 29 SC adalimumab–treated patients (9.9% [95% CI 6.5%, 13.3%]).

### Local injection site reactions

Local injection site reactions occurred in significantly fewer patients in the SC abatacept group than in the SC adalimumab group (12 [3.8%] versus 30 [9.1%] [95% CI −9.13%, −1.62%]; *P* = 0.006) (Table 2). The intensity of the injection site reactions in SC abatacept–treated patients was generally mild, with only 1 moderate reaction. Injection site reactions in the SC adalimumab group were mostly mild, but 6 were moderate and 1 was severe. There was a greater diversity in the type of injection site reactions reported in SC adalimumab–treated patients. No patients in the SC abatacept group discontinued treatment due to injection site reactions, while 3 patients discontinued treatment in the SC adalimumab group.

## DISCUSSION

In this first head-to-head comparative study from the AMPLE trial, we demonstrated the noninferiority of SC abatacept compared to SC adalimumab, each given in combination with background MTX, in RA patients with moderate-to-severe disease who were naive to treatment with bDMARDs and whose mean disease duration was <2 years. The primary outcome measure, the ACR20 response, was achieved by 64.8% of SC abatacept–treated patients and 63.4% of SC adalimumab–treated patients. Similar benefits in terms of treatment efficacy were seen across all disease activity measures, including the ACR50 and ACR70 improvement responses and the DAS28-CRP score. Both agents showed similar kinetics of clinical response and comparable inhibition of radiographic damage progression. These results are consistent with those seen in previous studies of abatacept and adalimumab (4–11). Overall, the rates of safety events were balanced, other than a significantly higher rate of local injection site reactions among SC adalimumab–treated patients.

Radiographic outcomes are a hallmark of any substantial RA trial. Measures of radiographic damage represent objective and quantifiable outcomes that are correlated with disease activity, long-term disability, and treatment benefit (25, 30, 31). This study provides the first radiographic data set for the SC abatacept formulation. The data showed similar and robust clinical benefits, in terms of improvements in both the erosion and joint space narrowing scores, in RA patients treated with either SC abatacept or SC adalimumab in combination with background MTX, as was also seen in earlier studies (5, 7). The radiographic nonprogression rates and overlapping cumulative probability curves demonstrate that inhibition of radiographic progression was comparable between the treatments and included most patients.

Safety outcomes in year 1 were mostly balanced, with some important differences. The rates and types of AEs reported, including infections and SAEs, were comparable between the treatment groups and were consistent with those previously reported in clinical trials of both agents (32–35), but led to higher rates of discontinuation in the adalimumab group. A greater number of SC adalimumab–treated patients developed seropositivity for ANAs or anti-dsDNA autoantibodies during the course of treatment, consistent with observations in prior studies (36). Nonserious autoimmune AEs, however, occurred more frequently in the SC abatacept treatment group. Local injection site reactions were significantly more frequent in SC adalimumab–treated patients, and were also qualitatively different and led to more discontinuations in the SC adalimumab group. Overall, the safety outcomes are not well suited for statistical comparison, and are more suited for descriptive metrics. The value of comparative safety increases with the period of exposure and frequency of the events. Comparative RA trials might prove more informative when the safety events observed in these trials reflect the findings of larger safety databases.

This study illustrates the advantages and limitations of performing head-to-head trials. A noninferiority design was chosen for this study because both therapies were expected to be equally efficacious in the main assessment (14, 15). Noninferiority trials serve to establish treatment parity and allow for comparison of other relevant, secondary outcomes. The main benefit of these trials is to provide the most relevant comparative and robust data for evidence-based decisions (1, 13, 37). Previously, physicians relied on indirect, statistics-based strategies, with their associated limitations, e.g., disparate populations (38–40). Studies that include comparator groups not powered for direct comparison afford more value, but still have limitations (9, 41). This head-to-head trial was designed and powered for direct comparison at 1 year. The second year of the AMPLE trial is designed to provide additional information in a controlled study. For comparative trials to be truly informative, they must enroll subjects who resemble those seen in clinical practice, and utilize therapeutic agents in a manner that reflects routine use (1, 14). Adalimumab is a TNFi, the most commonly used bDMARD class in RA. Its clinical profile in the present study population is well established, and it is therefore an appropriate comparator choice.

The inability to properly mask the commercially acquired comparator agent, adalimumab, which has unique visual proprietary characteristics, is a limitation of the study. This prevented us from conducting a classic double-blind study, since patients were not blinded with regard to treatment allocation. It was neither feasible to require weekly visits for blinded injections for 2 years nor practical for a home visit nurse to administer the injections. The study did utilize an investigator-blind strategy, which mitigates this potential weakness. The consistent findings across both patient- and physician-reported outcomes and the objective radiographic outcomes support the integrity of the investigator-blind approach utilized in the AMPLE trial.

Another potential area of concern was the selection of the DAS28-CRP as a measure of disease activity. The DAS28-CRP and the DAS28 using the ESR (DAS28-ESR) do not generate identical disease activity scores, and indeed application of the DAS28-CRP leads, frequently, to higher percentages of patients being classified as in remission. However, this is less relevant in the present study, since we compared the 2 treatments and the possible difference in rates of DAS28-ESR–defined remission, and rates of DAS28-CRP–defined remission would apply to both drugs.

Current use of bDMARDs in the treatment of RA is not based on head-to-head comparative data. This study demonstrates that treatment of RA patients with agents that have different mechanisms of action, either T cell modulation or TNF inhibition, will lead to comparable clinical benefit, suggesting that these 2 agents should be considered equally for the treatment of RA patients who have an inadequate response to MTX. These findings from the AMPLE study demonstrate the value of comparative trials and should serve as a blueprint for future head-to-head trials in RA.

## AUTHOR CONTRIBUTIONS

All authors were involved in drafting the article or revising it critically for important intellectual content, and all authors approved the final version to be published. Dr. Weinblatt had full access to all of the data in the study and takes responsibility for the integrity of the data and the accuracy of the data analysis.

**Study conception and design.** Weinblatt, Schiff, Zhao, Maldonado, Fleischmann.

**Acquisition of data.** Valente, van der Heijde, Citera, Zhao, Maldonado, Fleischmann.

**Analysis and interpretation of data.** Weinblatt, Schiff, Valente, van der Heijde, Citera, Zhao, Maldonado, Fleischmann.

## ROLE OF THE STUDY SPONSOR

Bristol-Myers Squibb funded the study. The study protocol was jointly developed by the academic authors and Bristol-Myers Squibb. Professional medical writing and editorial assistance was provided by Anu Santhanagopal, PhD, of Bristol-Myers Squibb. Publication of this article was not contingent upon approval by Bristol-Myers Squibb.
